# Electrical and Capacitive Response of Hydrogel Solid-Like Electrolytes for Supercapacitors

**DOI:** 10.3390/polym13081337

**Published:** 2021-04-19

**Authors:** Guillem Ruano, José I. Iribarren, Maria M. Pérez-Madrigal, Juan Torras, Carlos Alemán

**Affiliations:** 1 Departament d’Enginyeria Química, Universitat Politècnica de Catalunya, Campus Diagonal Besòs (EEBE), C/Eduard Maristany, 10-14, 08019 Barcelona, Spain; guillem.ruano@upc.edu (G.R.); jose.iribarren@upc.edu (J.I.I.); 2 Barcelona Research Center for Multiscale Science and Engineering, Universitat Politècnica de Catalunya, Campus Diagonal Besòs (EEBE), C/Eduard Maristany, 10-14, 08019 Barcelona, Spain; 3 Institute for Bioengineering of Catalonia (IBEC), The Barcelona Institute of Science and Technology, Baldiri Reixac 10-12, 08028 Barcelona, Spain

**Keywords:** flexible hydrogels, supercapacitor, biopolymers, electrochemical impedance spectroscopy

## Abstract

Flexible hydrogels are attracting significant interest as solid-like electrolytes for energy storage devices, especially for supercapacitors, because of their lightweight and anti-deformation features. Here, we present a comparative study of four ionic conductive hydrogels derived from biopolymers and doped with 0.1 M NaCl. More specifically, such hydrogels are constituted by κ-carrageenan (κC), carboxymethyl cellulose (CMC), poly-γ-glutamic acid (PGGA) or a phenylalanine-containing polyesteramide (PEA). After examining the morphology and the swelling ratio of the four hydrogels, which varies between 483% and 2356%, their electrical and capacitive behaviors were examined using electrochemical impedance spectroscopy. Measurements were conducted on devices where a hydrogel film was sandwiched between two identical poly(3,4-ethylenedioxythiophene) electrodes. The bulk conductivity of the prepared doped hydrogels is 76, 48, 36 and 34 mS/cm for PEA, PGGA, κC and CMC, respectively. Overall, the polyesteramide hydrogel exhibits the most adequate properties (i.e., low electrical resistance and high capacitance) to be used as semi-solid electrolyte for supercapacitors, which has been attributed to its distinctive structure based on the homogeneous and abundant distribution of both micro- and nanopores. Indeed, the morphology of the polyestermide hydrogel reduces the hydrogel resistance, enhances the transport of ions, and results in a better interfacial contact between the electrodes and solid electrolyte. The correlation between the supercapacitor performance and the hydrogel porous morphology is presented as an important design feature for the next generation of light and flexible energy storage devices for wearable electronics.

## 1. Introduction

In the last decade, organic electronics has been considered as a key technology for portable and wearable energy-storage devices, such as batteries and supercapacitors, that require flexibility, and lightweight and anti-deformation properties [1,2,3,4,5]. Although batteries can store a high amount of specific energy, they exhibit low power-handling capabilities, delivering electricity at low current densities [6,7]. Instead, supercapacitors provide high specific power and efficiency, as well as a higher cyclic durability than batteries [8,9].

In recent years, progress in the development of flexible supercapacitors has been mainly focused on the manufacture of flexible electrodes, which include not only flexible substrates coated with a thin layer of electrochemically active materials, [10,11] but also free-standing conducting carbon based materials, such as carbon nanotubes- and graphene-containing materials [12,13,14,15,16]. However, the combination of such electrodes with conventional liquid electrolytes presents serious limitations since the undesired leakage of harmful liquid and the dislocation of electrodes occur when the device is repeatedly bended or compressed. Furthermore, the incompressible behavior of liquid electrolytes hinders the mechanical integrity of the devices. In order to overcome those drawbacks, the substitution of liquid electrolytes by hydrogel solid-like electrolytes has appeared as a simple strategy able to prevent the above-mentioned inconvenience under harsh mechanical conditions.

A hydrogel consists of a polymer network (i.e., with physical or chemical crosslinks) and an aqueous solution. The polymer makes the hydrogel an elastic solid, while the aqueous solution containing a salt (e.g., NaCl) makes it an ionic conductor. The mesh size of the polymer network is larger than the size of water molecules and ions coming from the salt, thus allowing water molecules in the hydrogel to maintain the same chemical and physical properties as in a liquid-state solution [17]. Although currently, polyvinyl alcohol (PVA)-based gels are the most widely used electrolytes for solid-state supercapacitors, [1,18] biopolymer-derived systems are gaining increasing attention. For example, polysaccharides, [19,20,21,22,23] proteins and polypeptides, [24,25,26,27] and even synthetic polymers incorporating biological units, such as polyesteramides, [28,29] have been used to prepare hydrogels as solid-like electrolytes for manufacturing bioinspired supercapacitors. Indeed, their electrochemical response has been well studied by cyclic voltammetry and galvanostatic charge-discharge cycles; [1,18,19,20,21,22,23,24,25,26,27,28,29] however, their ionic conductivity and capacitive properties remain unknown in many cases. It is worth noting that materials to be applied as an electrolyte must exhibit high ionic conductivity, in the order of ~10^−3^ S/cm at room temperature [30]. Although this condition is fulfilled by synthetic hydrogels, such as polyethylene oxide (PEO)-based hydrogel electrolytes with 30% wt. KOH (~10^−3^ S/cm), [31] PVA blended with PEO (~10^−2^ S/cm), [32] and potassium polyacrylate (PAAK; ~0.3 S/cm), [33] the ionic conductivity of many biopolymeric hydrogels is yet to be determined.

Hence, in this work, we investigated the conductive and capacitive properties of different biopolymeric hydrogels using electrochemical impedance spectroscopy (EIS). More specifically, hydrogels from the following four biopolymers were prepared: (1) κ-carrageenan (κC); [23] (2) carboxymethyl cellulose sodium salt (CMC); [22] (3) poly-γ-glutamic acid (PGGA); [27] and (4) polyesteramide containing phenylalanine, butenediol and fumarate (PEA). [29] By applying EIS, we aimed to obtain additional information on their supercapacitor performance and thus, fully characterize these hydrogel-based systems to act as solid-like electrolytes. To that end, all prepared hydrogels were doped with 0.1 M NaCl. After evaluating their morphology and swelling ratio, data derived from EIS studies, including the electrical equivalent circuits (EECs) were obtained and discussed. Overall, the suitability of these biopolymer-derived systems in organic electronics was confirmed. Concretely, the PEA hydrogel, which contains biological units, exhibits the most adequate properties—in terms of electrical resistance, capacitance and interfacial contact—to be used as semi-solid electrolyte for supercapacitors.

## 2. Methods

### 2.1. Materials

All reagents were used as purchased without further purification. κC sulfated plant polysaccharide (22,048, 5–25 mPa·s, 0.3% in H_2_O (25 °C)), NaCMC with high viscosity (1500–3000 cP, 1 % in H_2_O at 25 °C), cystamine dihydrochloride (≥98.0%), 1-[3-(dimethylamino)propyl]-3-ethylcarbodiimide methiodide (EDC methiodine), citric acid (99%), sodium azide (NaN_3_ ≥ 99.5%), L-phenylalanine (reagent grade, 98%), *p*-toluenesulfonic acid monohydrate (ACS reagent, 98.5%), *cis*-2-butene-1,4-diol (97%), toluene (99.8%), fumaryl chloride (95%), acetone (HPLC, 99.9%), acryloyl chloride (97%), *N*,*N*-dimethylacetamide anhydrous, 1-butanol (ACS reagent, 99.4%), *n*-hexane (reagent grade) and 2-hydroxy-4′-(2-hydroxyethoxy)-2-methylpropiophenone (Irgacure) were purchased from Sigma-Aldrich (Merck KGaA, Darmstadt, Germany). Free-acid PGGA (from *Bacillus subtilis*) with average molecular weight (M_w_) of 350,000 was purchased from Wako Chemicals GmbH (Neuss, Germany). Poly(ethylene glycol) (*M_n_* = 10,000 g/mol) and ethyl acetate were purchased from Honeywell Fluka™ (Fisher Scientific SL, Madrid, Spain). Dimethyl sulfoxide (DMSO, Analytical reagent) was purchased from Fisher Scientific SL (Madrid, Spain).

### 2.2. Synthesis

The synthesis of the four studied hydrogels has already been reported [28,34,35,36] and, therefore, a brief description of the employed procedures is only provided.

#### 2.2.1. κ-Carrageenan (κC) Hydrogel

κC has one ester sulfate group in the repeating unit, which forms strong and rigid gels in the presence of K^+^. In this work, κC was dissolved in water at 2% *w*/*v* at ca. 75–80 °C. Then, the corresponding volume of 1 M KCl (10% *v*/*v*) was added, and the solution stirred vigorously. Finally, the hydrogel was obtained by cooling the hot κC aqueous solution to room temperature for several hours, enabling a 3D network in which polymeric segments form junction points cross-linked by K^+^ [34]. The κC hydrogel was washed with distilled water three times.

#### 2.2.2. Carboxymethyl Cellulose Sodium Salt (CMC) Hydrogel

CMC (10% wt.) was mixed with water using, firstly, a high speeded magnet mixer and later, as the viscosity increased, manually with a glass rod. Then, the resulting CMC paste was processed in small pieces with a hydraulic press (10 Pa, 1 min), which were kept at 4 °C prior to the formation of the hydrogel. CMC polymeric chains were cross-linked by immersing the pieces into 1.5 M citric acid solution for 24 h at room temperature under slight shaking (80 rpm). [35] The excess of citric acid was removed by washing the pieces four times with distilled water.

#### 2.2.3. Poly-γ-Glutamic Acid (PGGA) Hydrogel

PGGA hydrogel (1 mL) was prepared by dissolving PGGA and EDC in 750 µL of sodium hydrogen carbonate solution (0.5 M) at 4 °C under magnetic stirring. Then, cystamine dihydrochloride, previously dissolved in 0.25 mL sodium hydrogen carbonate solution (0.5 M), was added to the solution and mixed for 2–3 min. The PGGA/EDC/cystamine molar ratio was 5/5/2.5. The final solution was mixed with a magnetic stirrer, and then poured into glass molds. The solution was let to gel at room temperature for one hour [36]. In order to remove any compound in excess, the resulting hydrogel was washed with distilled water three times.

#### 2.2.4. Polyesteramide (PEA) Hydrogel

The synthesis of the di-*p*-toluenesulfonic acid salt of L-phenylalanine butene 1,4-diester and di-*p*-nitrophenyl fumarate monomers, as well as their polycondensation to produce UPEA chains with C=C double bonds in the backbone, were conducted using the procedure described by Katsarava and co-workers [37]. Functionalized polyethylene glycol, which was used as crosslinker, was prepared using triethylamine and acryloil chloride, as reported in recent work [28]. Then, a 1:4 UPEA:crosslinker (*w*/*w*) mixture was dissolved in dimethylacetamide for the reticulation reaction, which was conducted by adding the photoinitiator irgacure 2959 (5% wt.) and exposing the solution to an UV lamp (230 V, 0.8 A) for 4 h at room temperature. The resulting hydrogel was washed with distilled water, which was periodically replaced for 3 days.

### 2.3. Characterization

Infrared absorption spectra were recorded with a Fourier Transform FTIR 4100 Jasco spectrometer equipped with a Specac Model MKII Golden Gate attenuated total reflection (ATR) cell and a heated Diamond ATR.

Scanning electron microscopy (SEM) studies were performed using a Focused Ion Beam Zeiss Neon40 scanning electron microscope equipped with an energy dispersive X-ray (EDX) spectroscopy system and operating at 5 kV. Prior to SEM observation, samples were lyophilized. All samples were sputter-coated with a thin carbon layer using a K950X Turbo Evaporator to prevent electron charging problems. Pore size was determined from the SEM images using the software SmartTIFF (v1.0.1.2.).

The swelling ratio (*SR*, %) of the prepared hydrogels was determined according to

(1) $$SR=\frac{w_{W}-w_{D}}{w_{D}}$$

where *w_W_* is the weight of the hydrogels as prepared (after the washing step) and *w_D_* is the weight of the hydrogel after freeze-drying (dried hydrogel).

Electrochemical impedance spectroscopy (EIS) diagrams were taken at open circuit (OCP) over the frequency range of 100 kHz to 10 Hz with potential amplitude of 0.05 V using an AUTOLAB-302N potentiostat/galvanostat. All experiments were performed at room temperature using a hydrogel as solid electrolyte sandwiched between two poly(3,4-ethylenedioythiophene) (PEDOT) electrodes.

PEDOT electrodes were prepared by chronoamperometry (CA) using a constant potential of 1.40 V under nitrogen atmosphere and at room temperature. A three-electrode one-compartment cell was filled with 50 mL of acetonitrile containing EDOT monomer (10 mM) and LiClO_4_ (0.1 M) as supporting electrolyte. Stainless Steel AISI 316 sheets with an area of 6 cm^2^ were employed as working and counter electrodes. The reference electrode was an Ag|AgCl electrode containing a KCl saturated aqueous solution (E^0^ = 0.222 V vs. standard hydrogen electrode at 25 °C). The polymerization time (θ) was adjusted to obtain PEDOT electrodes with a polymerization charge of 2.67 C (445 mC/cm^2^).

After data collection, EIS results were then processed and fitted to an electrical equivalent circuit (EEC). The percentage error associated with each circuit element was acceptably low (see next section).

## 3. Results and Discussion

**Figure 1:**
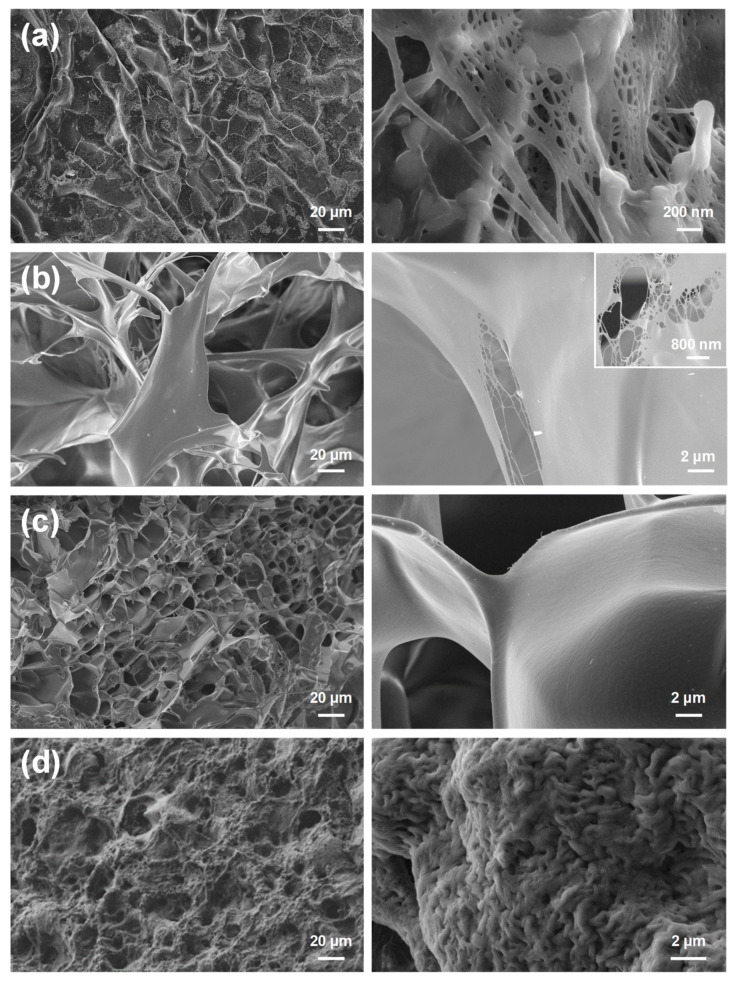
SEM micrographs of (**a**) κC, (**b**) CMC, (**c**) PGGA and (**d**) PEA hydrogels: General view (low magnification images, left) and details (high magnification images, right).

The successful preparation of the κC, CMC, PGGA and PEA hydrogels was confirmed by FTIR. The chemical structures derived from the recorded spectra (not shown) were fully consistent with those reported in previous works [22,23,27,29]. From a mechanical point of view, all four systems were flexible and deformable, displaying excellent stability and robustness to keep the electrodes at their positions. Before running EIS analyses, the morphology (Figure 1) and SR (Equation (1), Table 1) of the four hydrogels were examined.

SEM characterization was conducted to determine the morphology of the hydrogel networks and understand the correlation between pore size and the supercapacitor performance. Representative low and high magnification SEM micrographs of the four studied hydrogels are shown in Figure 1. In spite of their morphological differences, pores can be easily identified in the low magnification micrographs of all samples. Although κC, CMC and PGGA hydrogels present well defined pores with average size of 26 ± 9, 52 ± 14 and 19 ± 8 µm, respectively, the structure of κC is clearly less open than those of CMC and PGGA. Thus, the latter hydrogels show micropores similar to those typically found in honey-comb structures, whereas κC exhibits more compact and less interconnected microporous. In addition to such micrometric pores, representative high magnification micrographs reveal the existence of regions with nanometric pores, which are heterogeneously distributed and are expected to participate in the ion diffusion process. On the other hand, although PEA hydrogel exhibits a more compact structure, micrometric pores with an average size 14 ± 10 μm are clearly identified. Another remarkable difference between PEA and the other three hydrogels is the existence of nanometric pores (534 ± 118 nm) homogeneously distributed throughout the whole surface, which is evidenced in the high resolution SEM micrograph. Therefore, both κC and PEA hydrogels display a more densely packed network, which is expected to affect the conductive and capacitive features of the resulting device.

Swelling measurements gave us additional information about the porous structure of the hydrogels. Indeed, high SR values indicate that the swellable hydrogels are able to hold large amounts of water. In general, the amount of swelling depends on several factors, such as chemical structure, composition, crosslinking density, solvent, among others. Hence, although a porous structure was observed for the four systems, the different natures of the hydrogels could influence the swelling response. The SR of the four studied hydrogels varies as follows: PGGA < κC << PEA << CMC. It is worth noting that CMC shows the largest micropores (Table 1), which explains the very high SR of this hydrogel (SR = 2356 ± 240%). Instead, the average size of the micropores in PEA is comparable to that of PGGA, its high SR (SR = 1416 ± 300%) being attributed to the homogeneous and abundant distribution of nanopores on the whole surface.

**Figure 2:**
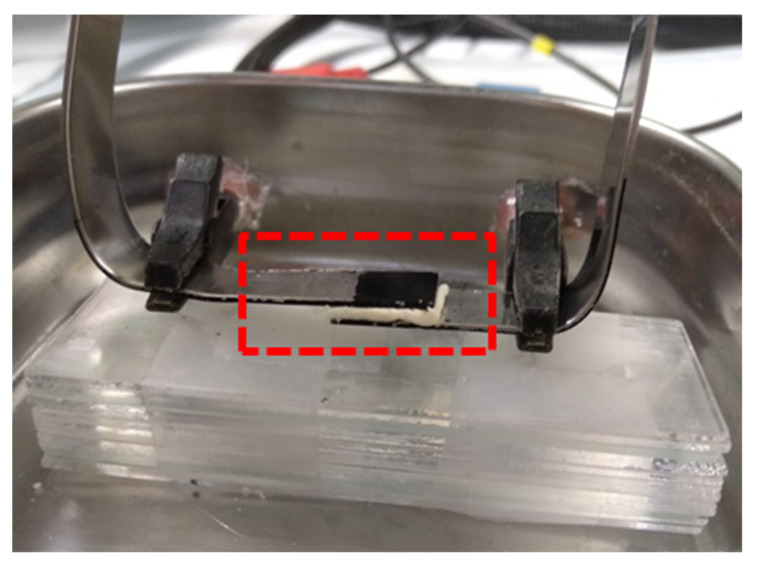
Sandwiched configuration used to perform EIS measurements on κC, CMC, PGGA and PEA hydrogels. The hydrogel separating the two PEDOT electrodes are marked by the read box.

EIS was employed to investigate the electrical and capacitive performance of κC, CMC, PGGA and PEA hydrogels, which were previously doped by soaking them in 0.1 M NaCl solution prepared with distilled water for 24 h. Then, hydrogels were cut in square cuboids of dimensions 0.5 × 0.5 × 0.1 cm^3^. EIS measurements were conducted at room temperature using a sandwiched configuration, in which each hydrogel piece was arranged separating two PEDOT electrodes at a distance of 0.1 cm (Figure 2). PEDOT electrodes were prepared as described in the Methods section.

**Figure 3:**
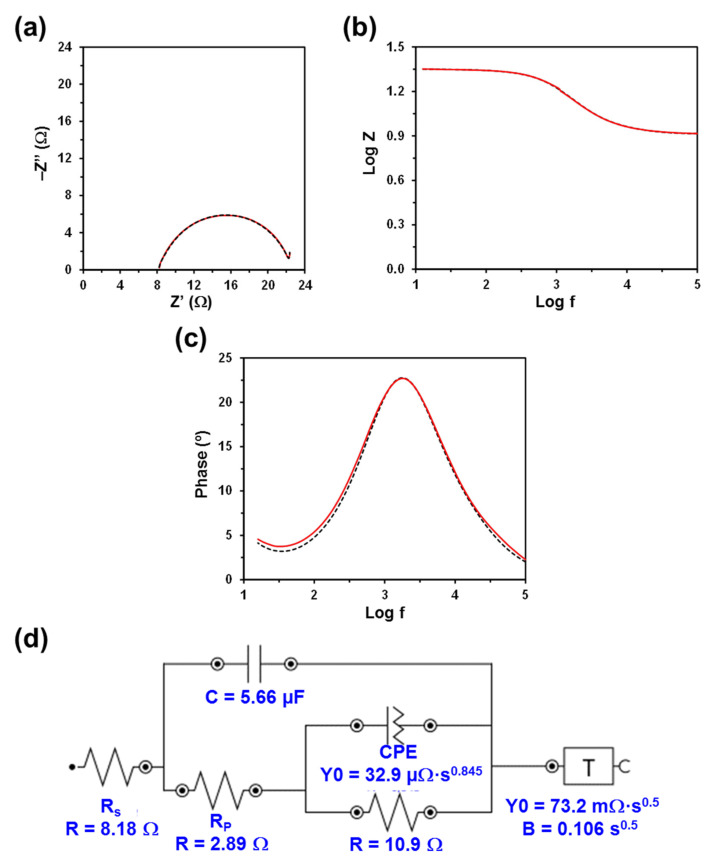
Measured and fitted EIS spectra (solid red line and dashed black line, respectively) for the κC hydrogel sandwiched between two PEDOT electrodes: (**a**) Nyquist plot; (**b**) impedance Bode plot; and (**c**) phase Bode plot. (**d**) EEC model used for numerical fitting of the EIS data. Numerical results from fitting the spectra are displayed for all the elements of the EEC.

**Figure 4:**
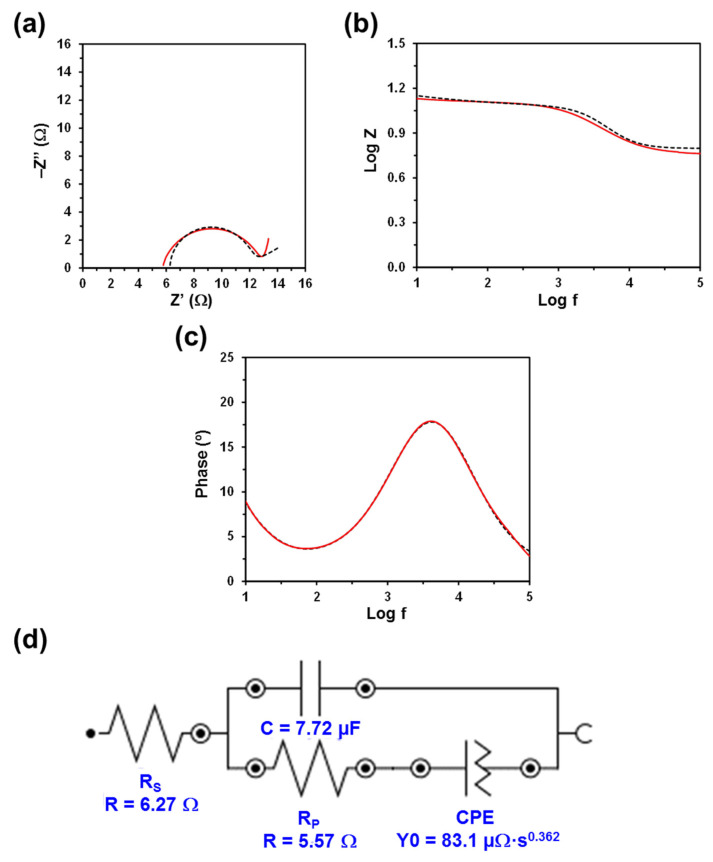
Measured and fitted EIS spectra (solid red line and dashed black line, respectively) for the CMC hydrogel sandwiched between two PEDOT electrodes: (**a**) Nyquist plot; (**b**) impedance Bode plot; and (**c**) phase Bode plot. (**d**) EEC model used for numerical fitting of the EIS data. Numerical results from fitting the spectra are displayed for all the elements of the EEC.

**Figure 5:**
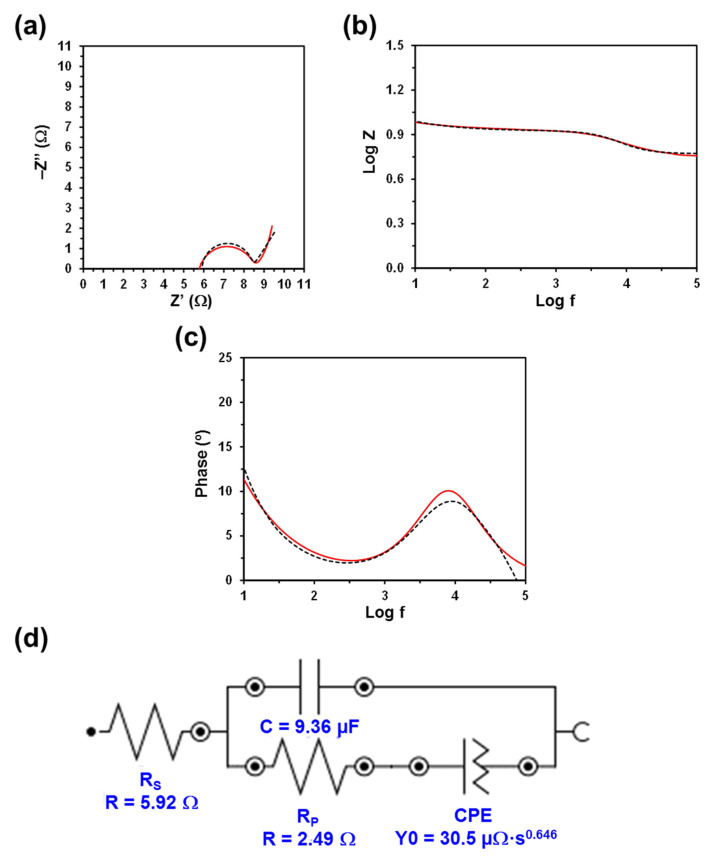
Measured and fitted EIS spectra (solid red line and dashed black line, respectively) for the PGGA hydrogel sandwiched between two PEDOT electrodes: (**a**) Nyquist plot; (**b**) impedance Bode plot; and (**c**) phase Bode plot. (**d**) EEC model used for numerical fitting of the EIS data. Numerical results from fitting the spectra are displayed for all the elements of the EEC.

**Figure 6:**
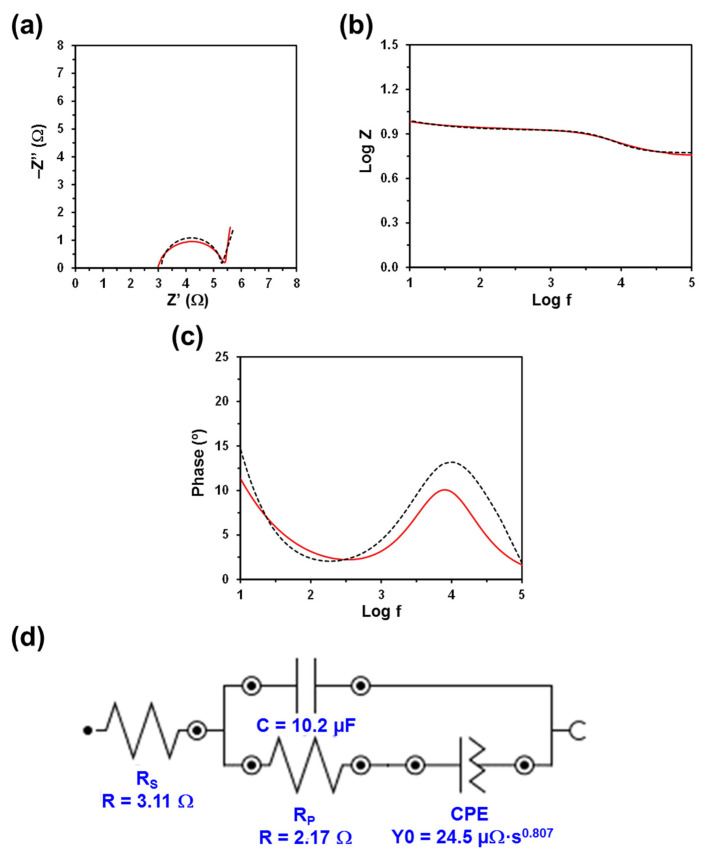
Measured and fitted EIS spectra (solid red line and dashed black line, respectively) for the PEA hydrogel sandwiched between two PEDOT electrodes: (**a**) Nyquist plot; (**b**) impedance Bode plot; and (**c**) phase Bode plot. (**d**) EEC model used for numerical fitting of the EIS data. Numerical results from fitting the spectra are displayed for all the elements of the EEC.

The Nyquist and Bode plots, as well as the electric equivalent circuit (EEC) derived from such diagrams, are displayed in Figure 3, Figure 4, Figure 5 and Figure 6. The quality of the experimental data fitted to the EEC was evaluated to estimate the percentage error associated with each circuit element, being comprised between 0.1% and 6.2% for κC, 0.3% and 7.1% for CMC, 0.3% and 5.2% for PGGA, and 0.3% and 5.1% for PEA. The EEC obtained for CMC, PGGA and PEA are identical (Figure 4, Figure 5 and Figure 6), while κC exhibits a more complex ECC with two additional elements (Figure 3).

The measured Nyquist plots, in which the real part of the impedance (Z) is plotted against the imaginary part (Z’), show a semicircle in all cases. The starting point of each curve (i.e., the intercept of the curve with the real Z-axis in the high frequency region) indicates the electrolyte resistance (R_S_), [38] that is, the resistance of the doping solution inside the hydrogel pore. R_S_ only depends on the ionic concentration, type of ions, temperature and the geometry of the area in which the current is carried (i.e., hydrogel mesh size). Considering that in this work all hydrogels were soaked in a 0.1 M NaCl electrolytic solution and that the temperature was very similar in all experiments, the small variation expected among the different analyzed systems should be attributed to the different concentration of NaCl inside the hydrogels (i.e., the doping capacity of the hydrogels) and the morphology of the hydrogel (i.e., mesh size), which may lead to a nonuniform ion concentration in the electrolyte. Thus, the lower the concentration and the ion mobility (i.e., more restricted by the morphology) in the hydrogel, the higher the R_S_ value will be.

Although the R_S_ values displayed in Figure 3, Figure 4, Figure 5 and Figure 6 are of the same order of magnitude, they increase as follows: PEA (3.11 Ω) < PGGA (5.92 Ω) < CMC (6.27 Ω) < κC (8.18 Ω). κC exhibited the most compact structure (Figure 1a) and, therefore, the largest area, which affected negatively the entrance of ions. Both CMC and PGGA exhibit pseudo-honeycomb structures with very similar R_S_ values, which are intermediate between those of κC and PEA. Finally, the low R_S_ value obtained for PEA is ascribed to its different microstructure: open interconnected pores surrounded by regions with compact morphology and homogeneously distributed throughout the surface following a bimodal distribution (Figure 1d). This feature, which resembles that proposed by Wang et al. at low temperature, [39] in addition to other factors, such as tortuosity, dead-end pores or the orientation of the microstructure, [40] might affect ion mobility inside the hydrogel. Overall, the resistivity of the doped hydrogel increases with R_S_. Thus, when the solution resistance increases, this means that the resistivity of the electrolyte solution increases and the pore area (solid phase area) decreases.

The diameter of the semicircle corresponds to the charge transfer resistance (R_P_) at the PEDOT electrode/hydrogel interface, also known as the interface reaction resistance [38]. Specifically, this element accounts for the ionic resistance (electrolyte resistance in the porous structure of the electrolyte) and the electronic resistance, which comprises the intrinsic resistance of the electrode material and the contact resistance between the active layer and the current collector. In all cases, the R_P_, which includes the resistance of the hydrogel and the PEDOT electrodes, is slightly lower than the R_S_. The values R_P_ obtained for κC, PGGA and PEA are similar (i.e., 2.89, 2.49 and 2.17 Ω, respectively) and lower than that of CMC (5.57 Ω), which indicates that the former hydrogels present better interfacial contact between the electrodes and the solid-like electrolyte. Indeed, the better the interfacial contact, the faster the ion transport and the lower the interfacial resistance.

The Bode diagrams, which represent the frequency response of impedance (Figure 3b and Figure 6b) and phase angle (Figure 3c and Figure 6c), display two time constants. The first is below 10^1^ Hz and is related to the hydrogel, whereas the second is around 10^3.2^, 10^3.6^, 10^3.9^ and 10^4.0^ Hz for κC, CMC, PGGA and PEA hydrogels, respectively. The second time constant has been associated with the interface between the PEDOT electrode and the hydrogel.

**Figure 7:**
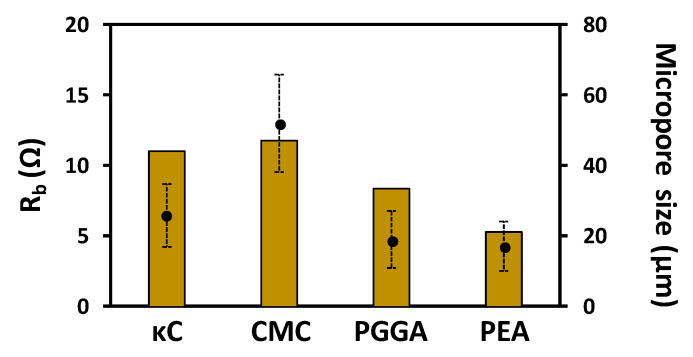
Relationship between pore size and the internal resistance for each of the hydrogel systems.

Based on their definition, the sum of the electrolyte resistance (R_S_) and the interfacial resistance (R_P_) can be interpreted as the internal resistance (R_b_ = R_S_ + R_P_), which corresponds to the bulk resistance of the whole device [38]. The values of R_b_ increase as follows: PEA (5.28 Ω) < PGGA (8.41 Ω) < κC (11.07 Ω) < CMC (11.84 Ω). Overall, the conductive properties of the supercapacitor device based on PGGA and, especially, PEA are superior to those of κC and CMC, which we ascribe to their better interfacial contact and the hydrogel structure (Figure 7).

The bulk conductivity (σ) of the prepared doped hydrogels is 76, 48, 36 and 34 mS/cm for PEA, PGGA, κC and CMC, respectively. It is worth noting that the conductivity of these biohydrogels is comparable to those reported for PVA-containing hydrogels; for instance, PVA doped with H_3_PO_4_ or H_2_SO_4_ (11.6 or 7.1 mS/cm, respectively), [41,42] PVA doped with H_3_PO_4_ and 2-mercaptopyridine (22.6 mS/cm), [41] PVA doped with H_2_SO_4_ and indigo carmine or alizarin red S (20.3 or 33.1 mS/cm, respectively), [43] chemically crosslinked PVA-poly(ethylene glycol) (67.1 mS/cm) [44] and KCl doped boron cross-linked PVA (38 mS/cm). [45] Moreover, the ionic conductivity of a 0.1 M NaCl aqueous solution is ~20 mS/cm, a concentration of 1 M NaCl being required to obtain a value comparable to that of doped PEA hydrogel [46]. This feature evidences the important role of the hydrogel structure in the mobility of ions.

The EEC used to fit the experimental EIS results for CMC, PGGA and PEA corresponds to the Randles-type electrical equivalent circuit [47]. In addition to R_S_ and R_P_, the diagram shows two additional elements: (1) the capacitance C of the hydrogel in parallel with R_P_; and (2) the constant phase element (CPE), which is associated with the capacitance of the electrical double layer (C_dl_) on the electrode surface, in series with R_P_. In the case of κC, the CPE is in parallel with resistance and, in addition, it shows a tangent hyperbolic diffusion element (T), also named bounded Warburg, which is common for porous electrodes.

The perfect semicircle obtained in the Nyquist plot for all the studied systems indicates that the four doped hydrogels behave as ideal capacitors. Accordingly, the capacitance C, which is in parallel with R_P_, reflects their ability to store charge. The C values obtained for κC, CMC, PGGA and PEA are 5.56, 7.72. 9.36 and 10.2 µF, respectively. Thus, the amount of accumulated charge increases with decreasing R_S_, which evidences that the capacitance improves with the mobility and transport of ionic charge.

The C_dl_ is associated with the separation of charges at the electrode/electrolyte interface. The double layer capacitance was represented with a CPE, which describes a non-ideal capacitor when the phase angle is different from −90°, to model the heterogeneity of the samples. The CPE impedance is attributed to the distributed surface reactivity, surface heterogeneity, and roughness of the current and potential distribution, which in turn are related to the electrode geometry and the electrode porosity [48]. The CPE impedance has been expressed as

(2) $$Z_{\mathrm{CPE}}={\left[Y_{0}{\left(j\cdot \omega \right)}^{n}\right]}^{-1}$$

where *Y*_0_ is the admittance of an ideal capacitance and *n* is an empirical constant, ranging from 0 to 1. When *n* = 1, the CPE behaves as a pure capacitor and *Y*_0_ = 1/C, while the CPE behaves a pure resistor and *Y*_0_ = 1/R when *n* = 0. Furthermore, when *n* = 0.5, the CPE is associated with a diffusion process, being the equivalent of the so-called Warburg element (W) and *Y*_0_ = √2/W. The *n* values obtained for κC and PEA (*n* = 0.845 and 0.807, respectively) are close to 1, while those of CMC and PGGA (*n* = 0.362 and 0.646, respectively) are close to 0.5.

The *T* diffusion element found in the EEC of κC, which is characteristic of films that contain a fixed amount of electroactive material, [49,50] in this case PEDOT, appears when the electroactive material cannot be replenished once it has been consumed. The *T* element is characterized by two parameters, an “admittance” parameter, *Y*_0_, and a time constant parameter, *B* (units: sec^½^):

(3) $$Z_{T}\frac{1}{Y_{0}\sqrt{j\cdot \omega }}\cot h\left(B\cdot \sqrt{j\cdot \omega }\right)$$

The parameter *Y*_0_ is related to the diffusion coefficient for the mobile species within the film. In this case, the *T* element has been associated with the counter ions confined into the PEDOT film situated between the metal substrate and the κC solid electrolyte. This confinement, which may be due to the compact structure of the hydrogel, is also responsible for the apparition of a resistance element linked to the electrode.

Hydrogels based on biopolymer-derived networks have emerged as a green energy approach to produce solid-like electrolytes. The four polymeric systems studied, which have been successfully applied as supercapacitors, are presented as technology options for energy storage devices. From a fundamental aspect, the characterization by EIS of the ionic and capacitive properties allowed us to choose the most suitable option. Most importantly, the correlation between such performance and the hydrogel porous morphology can be used as a design tool for the next-generation innovative systems.

## 4. Conclusions

In summary, the doped polyesteramide hydrogel, which displays micropores, as well as nanometric pores, homogeneously distributed throughout the whole surface, presents better properties as a solid-like electrolyte than doped biohydrogels with pseudo-honeycomb and compact heterogeneous structures. Indeed, the polyesteramide hydrogel shows a low electrical resistance, a high capacitance and good interfacial contact with the electrode, thus meeting the electrical requirements of solid-like electrolytes for supercapacitors. The full characterization of the hydrogel solid-like electrolytes by EIS provides additional valuable data (i.e., ionic and capacitive properties) to select the most adequate system. In addition, the hydrogel morphology, which has a significant effect on the device performance, is required to be highly porous and open. The correct optimization of these parameters would improve the application of biopolymer-derived hydrogels in light and wearable flexible devices.

## Figures and Tables

**Table 1:** Diameter of the micropores (D), swelling ratio (SR) and resistance (R_p_) of the four studied hydrogels.

Hydrogel	D (µm)	SR (%)	R_S_ (Ω)	R_P_ (Ω)
κC	26 ± 9	1070 ± 122	8.18	2.89
CMC	52 ± 14	2356 ± 241	6.27	5.57
PGGA	19 ± 8	483 ± 39	5.92	2.49
PEA	17 ± 7	1416 ± 297	3.11	2.17

## Data Availability

The data presented in this study are available on request from the corresponding author.
